# Guidelines for the Management of Soft Tissue Sarcomas

**DOI:** 10.1155/2010/506182

**Published:** 2010-05-31

**Authors:** Robert Grimer, Ian Judson, David Peake, Beatrice Seddon

**Affiliations:** ^1^Royal Orthopaedic Hospital NHS Trust, Birmingham B31 2AP, UK; ^2^Sarcoma Unit, Royal Marsden Hospital, London SW3 6JJ, UK; ^3^The Cancer Centre, Queen Elizabeth Hospital, Birmingham B15 2TH, UK; ^4^Department of Oncology, University College London Hospital NHS Trust, London NW1 2PG, UK

## Abstract

These guidelines were drawn up following a consensus meeting of UK sarcoma specialists convened under the auspices of the British Sarcoma Group and are intended to provide a framework for the multidisciplinary care of patients with soft tissue sarcomas. The guidelines published by the European Society of Medical Oncology (ESMO) and the National Comprehensive Cancer Network (NCCN) were used as the basis for discussion and adapted according to UK clinical practice and local requirements. Note was also taken of the National Institute for Health and Clinical Excellence (NICE) improving outcomes guidance (IOG) for people with sarcoma and existing technology appraisals. The guidelines are not intended to challenge NICE guidance but discrepancies may exist where current guidance does not reflect an international standard of care owing to the ever-evolving nature of cancer treatment. It is acknowledged that these guidelines will require updating on a regular basis. An appendix lists the key recommendations which are summarised below. Any patient with a suspected soft tissue sarcoma should be referred to a diagnostic centre and managed by a specialist sarcoma multidisciplinary team. Surgical excision followed by post operative radiotherapy is the standard management of high grade limb sarcomas although occasionally amputation remains the only option. Pre-operative treatment with chemotherapy or radiotherapy should be considered for patients with borderline resectable tumours. Isolated limb perfusion may permit limb salvage in some cases where amputation is the only other option. Adjuvant chemotherapy is not routinely recommended but may be considered in certain specific situations. Regular follow up is recommended to assess local control and the development of metastatic disease. Single agent doxorubicin is the standard first line therapy for metastatic disease. Ifosfamide is an alternative if anthracyclines are contraindicated. Combination therapy may be considered in individual patients. Second line agents include ifosfamide, dacarbazine, trabectedin and the combination of gemcitabine + docetaxel. Surgical resection of local recurrence and pulmonary metastases should be considered in individual patients. There is specific guidance on the management of retroperitoneal and uterine sarcomas.

## 1. Introduction

### 1.1. Rationale and Objective of Guidelines

Soft tissue sarcomas (STS) are a relatively uncommon group of malignancies. Although over the past few years, there have been advances in the understanding of the pathology, clinical behaviour and the treatment of this heterogeneous group of tumours, there was a concern that there was often no recognised “standard of care” for these patients in the UK. In the US, the National Comprehensive Cancer Network (NCCN) soft tissue sarcoma guidelines are highly regarded [1] as are those developed by The European Society of Medical Oncology (ESMO), which have been recently updated [2]. Using these two documents as a framework, clinical management guidelines for patients with STS in the UK were drawn up at a consensus meeting convened under the auspices of the British Sarcoma Group (BSG).

These guidelines are not intended to be prescriptive, but aim to improve the quality of care for patients with STS by helping identify and inform the key decisions involved in their management.

### 1.2. Methods

The NCCN and ESMO guidelines together with the National Institute for Health and Clinical Excellence Improving Outcomes Guidance for people with sarcoma (NICE-IOG) [3] were used as the basis for discussion by the group, adding or omitting detail only where it was clearly agreed by the consensus, in relation to UK specific issues.

### 1.3. Scope of Guidelines

These recommendations apply principally to “adult type” soft tissue sarcomas arising from limbs and trunk and although, where appropriate, specific guidance is given according to histological subtype it is recognised that some tumours, for example, Ewing's sarcoma and embryonal and alveolar rhabdomyosarcoma require a different approach to management, and these are excluded from this guidance [4]. Recommendations on the management of retroperitoneal and uterine sarcomas are included separately. Gastrointestinal stromal tumours (GISTs) are subject to their own specific guidelines, and will not be covered here [5].

These guidelines focus on clinical effectiveness, giving a picture of what treatments a specialist sarcoma multidisciplinary team should have access to within the UK, subject to some flexibility to allow for evolving practice, but, they do not purport to employ the same detailed analysis of cost effectiveness as NICE. These Guidelines can be considered to represent a broad consensus in 2010. They will need updating as treatment evolves.

## 2. Epidemiology

Sarcomas are relatively uncommon tumours accounting for approximately 1% of all adult cancers [6]. They constitute a heterogeneous group of tumours of mesenchymal cell origin, often with a distinct age distribution, site of presentation, natural biological behaviour and prognosis. There are more than 50 separate histological subtypes divided into two broad categories: soft tissue sarcomas and sarcomas of bone.

Historically, because of the heterogeneity of this group of tumours, the true incidence has generally been under-reported. Recent estimates from the cancer networks suggest that about 3000 patients (all sarcomas) are diagnosed per annum in the UK, including sarcomas of the head and neck, gynaecological sarcomas and GIST that were either not accurately diagnosed in the past, or not captured by cancer registries. 

Soft tissue sarcomas may occur at any age, and although most common in middle aged and older adults, they are relatively more common in children and young adults, accounting for 7–10% of paediatric malignancies. They are an important cause of death in the 14–29 years age group [7–10]. Primary bone tumours are less common than soft tissue sarcomas, the incidence being approximately one fifth that of STS, although they represent a significant percentage of the cancer burden in young people under the age of 20 years. 

Approximately half of all STS patients with intermediate or high-grade tumours develop metastatic disease requiring systemic treatment [11]; the overall survival is approximately 50% at 5 years [12].

## 3. Aetiology

For the vast majority of cases, the aetiology is unknown, although there are certain genetic associations, such as the 10% lifetime risk of malignant peripheral nerve sheath tumour (MPNST) in individuals with familial neurofibromatosis, caused by mutations in the *NF1* gene [13]. Another example is the increased risk of sarcomas, both bone and soft tissue, in patients who have had a familial retinoblastoma, caused by inherited mutations in the *RB* gene [14]. Similarly, there is an increased risk of sarcomas, and other cancers in families with Li-Fraumeni syndrome who have inherited mutations in the *TP53* tumour suppressor gene [15].

## 4. Clinical Presentation

Due to the heterogeneous sites of origin of STS, it is difficult to clearly define the clinical features of the disease. However, any soft tissue lump exhibiting any of the following four clinical features should be considered to be malignant until proved otherwise [16]:

- increasing in size,
- size >5 cm,
- deep to the deep fascia,
- painful.

The more of these clinical features present, the greater the risk of malignancy with increasing size being the best individual indicator.

**Key Recommendations:**

- Any patient with a soft tissue mass that is increasing in size, has a size >5 cm or is deep to the deep fascia, whether or not it is painful, should be referred to a diagnostic centre with a suspected STS.

## 5. Investigation

### 5.1. Imaging

Any patient with a suspected STS should be referred to a diagnostic centre for triple assessment with clinical history, imaging and biopsy [3]. Whilst the preferred method of imaging is MRI, other options including computerised tomography (CT) or ultrasound may be appropriate depending on local expertise. Patients with a confirmed STS should be staged with a high resolution CT chest to exclude pulmonary metastases prior to definitive treatment, although plain chest X-ray may be acceptable in a minority of cases (e.g., the very elderly and those with small low grade lesions) [17]. CT abdomen and isotope bone scan are not recommended as routine staging investigations, however depending on the histological type and other clinical features, further staging assessments may be recommended (e.g., regional lymph node assessment for synovial sarcoma, clear cell sarcoma or epithelioid sarcoma; abdominal and pelvic CT scan for myxoid liposarcoma). Positron emission tomography (PET) scanning may be helpful in specific circumstances (e.g., prior to radical amputation following recurrent disease), but cannot at the present time be recommended as a routine staging investigation in patients with STS. Although some work has been done looking at tumour response using PET; this is currently still investigational.

### 5.2. Biopsy

The standard approach to diagnosis of a suspicious mass is core needle biopsy—several cores should be taken to maximise diagnostic yield. However, an incisional biopsy may be necessary on occasion and excisional biopsy may be the most practical option for superficial lesions <5 cm diameter. The biopsy should be planned in such a way that the biopsy tract can be safely removed at the time of definitive surgery to reduce the risk of seeding and should be performed either at a diagnostic clinic or by a sarcoma surgeon or radiologist following discussion with the surgeon. Fine needle aspiration (FNA) is not recommended as a primary diagnostic modality, although it may be useful in confirming disease recurrence.

### 5.3. Histology-Diagnosis

Histological diagnosis should be made according to the WHO Classification to determine the grade and stage of the tumour [18]. The grade should be provided in all cases where possible based on a recognised system. In Europe, the Fédération Nationale des Centres de Lutte Contre le Cancer (FNCLCC) grading system is generally used, which distinguishes three grades [19] (Table 1). Because of tumour heterogeneity, a core biopsy may not provide accurate information about grade. In addition, certain translocation-driven sarcomas have a relatively uniform cellular morphology and, as such, can be misleadingly scored as intermediate, rather than high grade. This is especially true for myxoid/round cell liposarcoma, for which a different grading system based on the percentage of round cells is often used. Additional information may be provided by radiological imaging but histology may be modified following assessment of the complete surgical resection specimen.

Pathologic diagnosis relies on morphology and immunohistochemistry. It should be complemented, for those diagnoses characterised by a chromosomal translocation, using molecular pathology, for example, fluorescent in-situ hybridisation (FISH) or reverse transcription polymerase chain reaction (RT-PCR), in particular when the clinical pathologic presentation is unusual, or the histological diagnosis is doubtful.

#### 5.3.1. Histology-Resection

The report on the resected specimen should comply with the recommendations for reporting of STS produced by the Royal College of Pathologists. The pathology report should include an appropriate description of tumour margins (i.e., whether they are intralesional, marginal, or wide, and include distance from surrounding tissues, or the presence of an anatomical barrier). The pathologic assessment of margins should be made in collaboration with the surgeon. Tumour size and grade should be documented.

#### 5.3.2. Classification of Margins

Four categories of surgical margin have been described histologically: intralesional, marginal, wide and radical [20].

**Intralesional**

Margin runs through tumour and therefore tumour remains.

**Marginal**

Surgical plane runs through pseudocapsule (reactive zone). The local recurrence rate is high because of tumour satellites in the reactive tissue.

**Wide**

Surgical plane is in normal tissue but in the same compartment as the tumour.The recurrence rate is low and is related only to skip lesions in the affected compartment.

**Radical**

The tumour is removed including affected compartments and there is a minimal risk of local recurrence.

If feasible, it is recommended that tumour samples should be collected and frozen both for future research and because new molecular pathological assessment techniques may become available later that could yield new information of direct value to the individual patient. Any tissue thus obtained is governed by the Human Tissue Authority; hence appropriate informed consent will need to be obtained from the patient. 

If preoperative treatment (such as neo-adjuvant chemotherapy and/or pre-operative radiotherapy) was undertaken, the pathology report should include an assessment of tumour response to therapy. However, in contrast with bone sarcomas, no validated system is currently available for STS, and the percentage of residual “viable cells” is not considered to have a specific prognostic significance.

### 5.4. Staging

Several different staging systems may be used in STS management. The most widely accepted STS classification system produced jointly by the American Joint Committee on Cancer (AJCC)/International Union against Cancer (UICC) includes information on both the grade and stage of the tumour (Table 2). 

The final stage grouping is thus as follows.

**Stage I**

1A = low grade, small, superficial or deep (G1-2, T1a-b, N0, M0).

1B = low grade, large, superficial (G1-2, T2a, N0, M0).

**Stage II**

IIA = low grade, large, deep (G1-2, T2b, N0, M0).

IIB = high grade, small, superficial or deep (G3-4, T1a-b, N0, M0).

IIC = high grade, large, superficial (G3-4, T2a, N0, M0).

**Stage III**

High grade, large, deep (G3-4, T2b, N0, M0).

**Stage IV**

Any Metastasis (Any G, Any T, N1 or M1).

**Key Recommendations:**

1. Magnetic resonance imaging (MRI) and core needle biopsy are recommended prior to definitive surgery.
2. Imaging of the thorax by CT scan for lung metastases should be done prior to radical treatment.
3. All patients with a suspected STS should be managed by a specialist Sarcoma MDT as specified in the NICE guidance.

## 6. Management

Soft tissue sarcomas are a diverse group of tumours and as our understanding of the differing natural history and response to treatment improves it is increasingly possible to tailor treatment according to the individual histology. The major therapeutic goals are long-term survival, avoidance of local recurrence, maximising function and minimising morbidity.

All patients should have their care managed by a formally constituted Sarcoma MDT. Decisions about surgery, chemotherapy, radiotherapy and the timing of all these modalities should be made by the Sarcoma MDT. For site specific STS (e.g., Gynaecological, head and neck) there should be a formal relationship between the sarcoma MDT and the site-specific MDT.

**Standard Therapy-Surgery**

Conservative surgery combined with post operative radiotherapy is standard treatment of limb and truncal tumours in the UK and achieves high rates of local control whilst maintaining optimal function. Radiotherapy may be avoided in patients with low grade tumours that have been completely resected or those with small, superficial high grade tumours resected with wide margins. Table 3 (adapted from the ESMO guidance) shows what is accepted as standard, individualised or investigational therapy [2].

### 6.1. Surgery

#### 6.1.1. Surgery for Localised Disease

Surgery is the standard treatment for all patients with adult-type, localised soft tissue sarcomas, and it should be performed by an appropriately trained surgeon. Evaluation of the resectability of a tumour is determined by the surgeon in consultation with the MDT, and depends on the tumour stage and the patient's co-morbidity. The primary aim of surgery is to completely excise the tumour with a margin of normal tissue. What constitutes an acceptable margin of normal tissue is not universally agreed but is commonly accepted as 1 cm soft tissue or equivalent (e.g., a layer of fascia). However, on occasion, anatomical constraints mean that a true wide resection is not possible without the sacrifice of critical anatomical structures (such as major nerves, or blood vessels) and in this situation, it may be acceptable to leave a planned microscopic positive surgical margin, having considered the risks of recurrence and morbidity of more radical surgery and having discussed these fully with the patient [22]. 

For patients who have undergone surgery and have an unplanned positive margin, re-excision should be undertaken if adequate margins can be achieved. Macroscopic residual disease imparts a poor prognosis and local control is unlikely to be achieved even with addition of post operative radiotherapy [23].

Patients with tumours that, because of size or position, are considered borderline resectable should be considered for down staging treatment (neo-adjuvant) with either chemotherapy or radiotherapy depending on histology of the tumour and the performance status of the patient (see below).

In some situations amputation may be the most appropriate surgical option to obtain local control and offer the best chance of cure. It is recognised that there is a group of low grade tumours which have a low risk of local recurrence and a low risk of metastasis and it is also appropriate to treat these by planned marginal excision. (e.g., atypical lipomatous tumours).

#### 6.1.2. Surgery in the Presence of Metastatic Disease

Surgical resection of the primary tumour may be considered appropriate as a palliative procedure in patients with metastatic disease, however radiotherapy or chemotherapy may be more appropriate and the decision must take into account factors such as the patient's symptoms (e.g., pain or fungation), co-morbidity, the expected morbidity of surgery, histological sub type and the extent of metastases and, of course, include a full discussion with the patient.

#### 6.1.3. Isolated Limb Perfusion

Isolated limb perfusion (ILP) is a valuable pre-operative technique for reducing the size of difficult but potentially resectable tumours in an extremity, where limb preservation may not otherwise be possible. ILP employs a locally high dose of chemotherapy (melphalan) and tumour necrosis alpha (TNF*α*) with hyperthermia localised to the affected limb using arterial and venous cannulation and a tourniquet. ILP has been shown to shrink peripheral tumours thus rendering them operable and should be considered in selected cases [24, 25]. ILP may also be considered for palliation. (Currently this service is only available for STS at the Royal Marsden Hospital in London and at the Beatson Cancer Centre in Glasgow, but it is more widely available for melanoma).

**Key Recommendations:**

- Surgery is the standard treatment for all patients with localised STS.
- For those patients with resectable disease, a wide excision is the standard surgical procedure.
- The definition of “wide” remains unclear but most would accept that an intact fascial layer or 1cm of normal tissue would be considered adequate.
- Where a wide excision is not possible due to anatomical constraints, a planned marginal excision plus radiotherapy may be an appropriate means of achieving tumour control while maintaining function.
- Occasionally amputation is the only surgical option to achieve adequate margins
- For patients with borderline resectable tumours, pre-operative treatment with chemotherapy or radiotherapy should be considered dependant on individual histology.
- Isolated Limb Perfusion may permit limb salvage in some cases where amputation is the only conventional surgical approach.

### 6.2. Radiotherapy

#### 6.2.1. Adjuvant Radiotherapy

Postoperative radiotherapy is considered to be the standard approach for nearly all intermediate or high-grade soft tissue sarcomas (see Table 3). This allows preservation of function with similar local control rates and survival to radical resection (i.e., compartmental excision/amputation) [26]. The majority of patients with low grade tumours will not require radiotherapy, however it should be considered for those with large, deep tumours that are incompletely resected, especially if adjacent to vital structures that could limit further surgery in the future. Patients who have undergone a compartmental resection or amputation do not require adjuvant irradiation assuming that the margins are clear.

The recommended postoperative radiation dose is 60–66 Gy in 1.8–2 Gy fractions [27]. A two-phase technique using a shrinking field is commonly employed; 50 Gy to the initial larger volume followed by 10–16 Gy to a smaller volume [28, 29]. This dose may need to be reduced if the field includes critical structures (for example the brachial plexus).

Attention is drawn to the VORTEX clinical trial in extremity soft tissue sarcomas [27]. This randomised clinical trial, which is currently recruiting in the UK, is comparing the standard two-phase conventional radiotherapy technique with a single phase to a smaller tissue volume, in an attempt to spare normal tissue and hence improve subsequent limb function without compromising local control.

#### 6.2.2. Neo-Adjuvant Radiotherapy

Pre-operative radiotherapy in limb sarcoma has been shown to be associated with increased postoperative complications compared to the standard postoperative treatment but less late toxicity (reflecting the lower pre-operative dose of 50 Gy compared with the postoperative dose of 66 Gy and a smaller treatment volume), with equivalent tumour control [30]. In the UK, pre-operative radiotherapy is not used routinely, but may be preferred in certain situations where the size of the radiation field required for post-operative treatment is likely to be associated with significant late morbidity, or when the tumour is of borderline operability and pre-operative radiotherapy is judged to be capable of rendering the tumour operable [31–33]. For certain radiosensitive histological subtypes, such as myxoid liposarcoma, pre-operative radiotherapy may be particularly advantageous, given the degree of tumour shrinkage that can be achieved. The standard regimen for pre-operative radiotherapy is 50 Gy, in 1.8–2 Gy fractions, followed by surgery approximately 6 weeks following completion of radiotherapy. Further radiotherapy (10–16 Gy) may be given post-operatively, if tumour margins are positive.

### 6.3. Chemotherapy

#### 6.3.1. Adjuvant Chemotherapy

The role of adjuvant chemotherapy remains unproven. Although currently not regarded as standard treatment in the UK, there is conflicting evidence, and it may be considered for individual patients with potentially chemosensitive subtypes on the basis that benefit cannot be excluded, even though it has not been proven (Table 4). It may be also considered in situations where local relapse would be untreatable or where adequate radiotherapy could not be administered owing to the sensitivity of adjacent structures, for example, spinal cord*. *A meta-analysis published in 1997 reported an improvement in local control and progression free survival, however although there was a trend towards an overall survival benefit this was not statistically significant [34]. These data have been supported by two more recent overviews [35, 36]. The latter did not use original trial data and included a large Italian trial which, when published in 2001, reported a significant survival benefit for adjuvant chemotherapy, however this has not been maintained with long-term follow up [37]. The preliminary data from the EORTC 62931, the largest trial of adjuvant chemotherapy for STS, has failed to demonstrate any benefit from chemotherapy in local control, progression free survival or overall survival in patients treated with adjuvant chemotherapy. Interestingly however it did demonstrate improved survival in both groups compared with previous studies. This was thought to be due to improved surgical techniques and increased use of adjuvant radiotherapy. The results of the final analyses are awaited with interest, together with an up-dated meta-analysis [38].

#### 6.3.2. Neo-Adjuvant Chemotherapy

Although there are limited data, pre-operative chemotherapy may be considered for those patients with large high grade tumours that are considered borderline resectable by the MDT. The age and any comorbidity of the patient together with the histology of the tumour need to be taken into account. There is a wide variation in chemosensitivity between different histological subtypes (Table 4) [39]. If the tumour is chemosensitive and adjacent to critical organs then chemotherapy may render the tumour suitable for conservative surgery whereas otherwise more radical surgery may have been necessary. For example response rates of ≥50% have been reported for synovial sarcoma [40, 41]. Similarly, myxoid liposarcomas are considered to be significantly more responsive than the majority of STS, although the evidence remains controversial [41, 42].

**Key Recommendations:**

- Postoperative radiotherapy is recommended following surgical resection of the primary tumour for the majority of patients with high-grade tumours, and for selected patients with large or marginally excised, low-grade tumours.
- The recommended dose for postoperative radiotherapy is 60–66 Gy; in 2 Gy per fraction
- Pre-operative radiotherapy is advantageous in terms of long-term functional outcome with equivalent rates of disease control when compared with postoperative radiotherapy. There is however an increased risk of postoperative wound complications.
- The recommended dose for pre-operative radiotherapy is 50 Gy, in 2 Gy per fraction.
- Adjuvant chemotherapy is not routinely recommended but could be considered in situations where it may contribute to local disease control, for example, where proximity to sensitive vital structures precludes giving an adequate dose of radiotherapy or in the case of an R1 resection and a further wide excision cannot be performed.

## 7. Prognosis and Follow Up

In common with other tumour sites there are no published data supporting specific follow-up protocols for STS patients, and there is an urgent need for research. Patients may be reassured by follow up and early detection of local relapse or pulmonary metastases may improve prognosis in some patients. Follow up should be discussed with the patient and the rationale and limitations explained.

Prognosis can be estimated by well established nomograms based on grade, depth, size and diagnosis as well as patient age [43]. Local recurrence is related to grade, margins of excision and use of radiotherapy. Whilst most events will arise in the first five years following diagnosis, low grade tumours in particular may relapse late. Follow up should be continued for a minimum of 8 years for high grade tumours and longer for low grade tumours. 

A recent survey on follow-up illustrated how varied the approach is at different centres, with no agreement on imaging, follow-up intervals or duration of follow-up [44]. Practices such as discharging low-grade tumour patients at five years when the evidence suggests they recur late are an issue. 

It is recommended that standard follow up consists of:

- clinical history,
- clinical examination to focus on local recurrence, with follow up using ultrasound or MRI where indicated by clinical suspicion,
- chest X-ray with subsequent CT used for investigating suspicious lesions.

In certain cases, this standard follow up can be extended or adapted according to individual risk or local practice. If a patient were deemed to be unfit either for pulmonary metastectomy or systemic treatment, then diagnosing metastases when the patient is asymptomatic has no purpose, so, for example, the chest X-ray can be dispensed with.

As per the ESMO guidelines [2], it is recommended that patients with intermediate/high grade tumours should be followed every 3-4 months in the first 2-3 years, then twice a year up to the fifth year, and once a year thereafter. It is recommended that patients with low grade tumours should be followed up every 4–6 months for 35 years, then annually thereafter. A further value of follow up is to monitor late adverse effects of treatment.

**Key Recommendations:**

- It is recommended that patients with intermediate or high grade sarcoma are followed up every 3-4 months for the first 2-3 years, then twice a year for up to 5 years, and annually thereafter.
- Patients with low-grade sarcoma should be followed up every 4–6 months for 3–5 years, then annually.
- Standard follow up practice should consist of:
  - investigation of any symptoms reported by the patient,
  - clinical examination to focus on local recurrence, with imaging follow up where indicated by clinical suspicion,
  - routine chest X-ray to exclude pulmonary metastases.

## 8. Treatment of Advanced Disease

In almost all cases the treatment intention for systemic disease is palliative. Approximately 50% of patients develop distant metastases and eventually die of disseminated disease; with a median survival of approximately 12 months from diagnosis of metastases [35, 45, 46].

The incidence of many of the individual sub-types of soft tissue sarcoma is too small to permit large-scale prospective randomised controlled trials. Accordingly data are gathered from a range of studies which include single-site and multi-site phase 2 trials, retrospective case series, sub-analyses of trials for which a range of histological subtypes are included and, for the rarer sub-types, individual case reports.

### 8.1. Palliative Chemotherapy

The management of advanced disease is complex; the approach to palliative treatment depends to some extent on whether or not symptoms are present. In order to achieve control of symptoms such as pain, or dyspnoea, it is necessary to achieve some degree of tumour shrinkage. An alternative approach to try and stabilise disease to delay onset of symptoms is equally acceptable. 

The published response rates for chemotherapy in STS vary enormously; from 10–50% depending on the drugs used, patient selection and histological subtype (Table 4). It has been established that good performance status, young age, and absence of liver metastases predict a good response to chemotherapy and improved survival time [46]. 

#### 8.1.1. Single-Agent Chemotherapy

As per the ESMO guidelines [2], standard first-line treatment in Europe is doxorubicin 75 mg/m^2^ 3 weekly. Duration of treatment depends on response but a maximum of 6 cycles is recommended because of the risk of cumulative cardiotoxicity. 

Whilst response rates may be <20% [47], around 45% patients are reported to derive “clinical benefit” [41]. However, in virtually all published studies the median survival of patients with metastatic sarcoma is ≤1 year. The overall poor outcome of these patients indicates the need for more effective agents.

The standard second-line treatment is ifosfamide—which is also used first line where anthracyclines are contra-indicated, for example in patients at high risk of cardiac complications or patients pre-treated with anthracyclines. Clinical trials have indicated a dose-response relationship and a dose of 9-10 g/m^2^ is recommended [48]. The response rate is in the region of 8%, although higher response rates have been observed with high-dose (>12 g/m^2^) and continuous infusion ifosfamide regimens in those patients pre-treated at standard doses [2, 49, 50].

#### 8.1.2. Combination Chemotherapy

Because of current uncertainty it is suggested that patients should be entered in clinical trials, for example, EORTC 62012, a trial investigating whether single agent doxorubicin 75 mg/m^2^ is equivalent to doxorubicin 75 mg/m^2^  + ifosfamide 10 g/m^2^ as first line chemotherapy [51]. A Cochrane review in 2006 concluded that combination regimens, compared with single-agent doxorubicin, given at conventional doses produced only marginal increases in response rates at the expense of increased toxic effects and with no improvements in overall survival [47]. Initial combination therapy may be considered appropriate for those patients with good performance status and no comorbidity and who would be expected to tolerate the increased toxicity, particularly if objective response is considered important for symptomatic improvement.

#### 8.1.3. Second-Line Chemotherapy

There is no recognised “standard” therapy following failure of doxorubicin and ifosfamide. Dacarbazine has activity as do a number of newer agents—gemcitabine, taxanes and trabectedin. The evidence for gemcitabine and docetaxel is greatest for uterine leiomyosarcomas, however, subsequent studies have demonstrated activity in soft tissue leiomyosarcoma and other tumour types [52, 53]. Likewise trabectedin, although licensed as second-line treatment for all soft tissue sarcomas, has been licensed on the basis of a randomised trial comparing two different treatment regimens in patients with predominantly leiomyosaroma and liposarcoma [54]. Other tumours, such as synovial sarcoma may also be sensitive. Pegylated liposomal doxorubicin and paclitaxel have been demonstrated to have significant activity in angiosarcoma [55, 56]. There is increasing evidence for the differential response to chemotherapy according to histological subtype and as knowledge increases it is expected that it will become increasingly possible to individualise treatment. For example; synovial sarcoma, leiomyosarcoma and myxoid liposarcoma are recognised as having higher response rates to chemotherapy and, conversely, alveolar soft part sarcoma, extraskeletal myxoid chondrosarcoma and solitary fibrous tumour are generally regarded as insensitive to chemotherapy and there are only occasional reports of responses in clear cell sarcoma.

A lot of these data are based on phase II trials; the decision to offer chemotherapy and choice of agent should be based on histology and toxicity profile following full discussion with the patient.

#### 8.1.4. Management of Local Recurrence

Local recurrences are often accompanied by metastatic disease and patients should be carefully staged for this. In the absence of overt metastatic disease every attempt should be made to regain local control by further surgery with adequate margins (wide or radical) and radiotherapy (if it has not been used previously). Amputation may be needed in selected cases.

#### 8.1.5. Surgery for Metastases

Following a diagnosis of lung or other metastases, the decision regarding metastasectomy should be based on disease-free period following primary surgery, total number of lesions per lung, tumour growth and evolution of disease [2]. The CT scan should be repeated in three months and if no new lesions have appeared and the disease is operable, surgery is usually recommended. Patients with metastatic disease should also have any local recurrence staged, either by CT or by PET scan, especially if surgical resection is being considered. While there are few data from prospective studies reporting survival of STS patients surgically treated for thoracic metastases, there are many long-term survivors (reported variously at 20–40% of all patients undergoing lung surgery) who have had the procedure.

**Key Recommendations:**

- Systemic treatments for the majority of advanced STS are not curative; median survival time is ≤12 months from diagnosis of metastases.
- Published chemotherapy response rates vary enormously; from 10–50% depending on the drugs used, patient selection, and tumour grade and histological subtype.
- Standard first-line treatment is single doxorubicin 75 mg/m^2^ every 3 weeks.
- Ifosfamide at a dose of 9–10 g/m^2^ may be used first line if anthracyclines are contraindicated and may be an option for second-line therapy, other options could be considered according to the histology.
- Although the combination of doxorubicin and ifosfamide has not been demonstrated to improve survival in comparison to single agent doxorubicin first line, response rates are higher and it may be considered in individual patients.
- Additional second-line agents include dacarbazine, trabectedin and the combination of gemcitabine + docetaxel. Reported response rates are in the range of 5–25%. Some appear more active in certain histological subtypes, particularly leiomyosarcomas and liposarcomas. The choice of agent depends on histology, toxicity profile and patient preference.
- Surgical resection of local recurrence and pulmonary metastases should be considered in individual patients although there are limited data on survival benefit.

## 9. Uterine Sarcomas

This group includes uterine leiomyosarcomas (ULMS), endometrial stromal sarcomas (ESS), malignant mixed mullerian tumour (MMMT) and undifferentiated endometrial sarcoma. As per the ESMO guidelines, standard treatment for all localised tumours is total abdominal hysterectomy (TAH), with some differences between the tumour types as described below [2].

**(i) Uterine Leiomyosarcoma**

ULMS, a cancer of the smooth muscle, accounts for 35–40% of all uterine sarcomas; LMS can affect women as young as their mid-20s, although most patients will be aged 50–60 years. Standard surgical management for non-metastatic disease is TAH +/− BSO. If wished the ovaries can be retained in pre-menopausal women. Lymphadenectomy is not routinely required in that incidence of lymph node involvement is <5%. Adjuvant pelvic radiotherapy for FIGO stage I and II disease is not recommended routinely. Adjuvant pelvic radiotherapy may be considered for selected high risk cases. Adjuvant chemotherapy is not routinely recommended. Chemotherapy for advanced/metastatic disease is as for STS at other sites, that is, doxorubicin as first line, ifosfamide as second line. Gemcitabine and docetaxel has demonstrated activity in the second-line setting in leiomyosarcoma. Trabectedin also seems to have useful activity in ULMS when used first or second line.

Oestrogen receptor (ER) and progesterone receptor (PgR) expression is seen in approximately 50% of patients with ULMS. Some low and intermediate grade tumours may be sensitive to oestrogen deprivation, although there are very few published data on this situation. It is however reasonable to look for receptor expression in those with relatively indolent tumours for which treatment with an aromatase inhibitor or a progestogen might be appropriate. However, receptor expression does not guarantee response to oestrogen-lowering therapy, and use of oestrogen-lowering therapies should be used with particular caution in patients with high grade rapidly progressing tumours.

**(ii) Endometrial Stromal Sarcoma**

This is the rarest form of uterine sarcoma and is a generally indolent disease with a long natural history. It was formally known as “low grade ESS”, on the basis of a mitotic count of less than 10 mitoses per 10 high powered fields, but is now termed simply ESS, with no distinction between “grade” (mitotic count is now recognised not to be prognostic). There is a high incidence of expression of oestrogen and progesterone receptors (ER and PgR), and evidence that these tumours are hormonally responsive. Standard surgical treatment is therefore total abdominal hysterectomy, with bilateral salpingo-ophorectomy (BSO) in pre-menopausal women, and hormone replacement therapy is contraindicated post-operatively. A single small study has suggested that adjuvant progestogens after surgery may improve outcome; routine use is not indicated but could be considered in high risk patients [57]. The role of adjuvant pelvic radiotherapy is uncertain given the paucity of published data. Recurrent or advanced disease may respond to anti-oestrogen therapy, with an aromatase inhibitor, or a progestogen. Tamoxifen is not recommended since its action may be pro-oestrogenic in this setting.

**(iii) Malignant Mixed Mullerian Tumour or Carcinosarcoma**

This mixed tumour type is the most common of the uterine sarcomas, accounting for 50% of cases predominantly in post-menopausal women. Although the phenotype is a mixture of carcinoma and sarcoma this is now generally regarded as an epithelial tumour, rather than a biclonal malignancy, and as such is not generally treated as sarcoma and systemic treatment is similar that used for ovarian and endometrial cancers. Tumours termed adenosarcoma, in which no malignant epithelial components can be identified, may behave differently and require sarcoma therapy.

**(iv) Undifferentiated Endometrial Sarcoma**

This disease entity was formally known as “high grade ESS”, but is now termed undifferentiated endometrial sarcoma. It is a highly aggressive anaplastic malignancy that does not express ER and PgR, with a poor prognosis even for early stage disease, and uncertain response to systemic treatment. Surgical management is TAH +/− BSO, with the option for adjuvant pelvic radiotherapy. Follow-up protocols and systemic treatment for advanced disease parallel those for adult-type soft tissue sarcomas [2]. Oestrogen-lowering therapies are generally not used.

There has been some reported success with cisplatin in treating uterine sarcomas but figures are distorted because of high numbers of carcinosarcoma/MMMT patients in the only large trial. No subset analysis has been offered, therefore this drug is not recommended.

**Key Recommendations:**

- Standard treatment for all localised uterine sarcomas is TAH. Lymphadenectomy is not routinely indicated.
- Oophorectomy is indicated for endometrial stromal sarcoma. These patients should not have post-operative hormone replacement therapy. Use of adjuvant anti-oestrogen therapy is not routinely indicated.
- Adjuvant pelvic radiotherapy has not been shown to improve local control or survival, and is not routinely indicated in FIGO stage I and II disease. However, it could be considered for selected high risk cases.
- Advanced/metastatic LMS and undifferentiated endometrial sarcoma are treated systemically with the same drugs as STS at other sites. Gemcitabine and docetaxel may be particularly useful for LMS.
- Advanced/metastatic ESS can be treated with anti-oestrogen therapy, with an aromatase inhibitor or progestogen.

## 10. Retroperitoneal Sarcomas

Although the principles of management of retroperitoneal sarcomas are similar to those for soft tissue tumours there are some important differences. Contrast-enhanced CT may be a valuable aid to diagnosis of well-differentiated/de-differentiated liposarcoma and in helping to plan surgery. Surgical margins are often more difficult to define as transcoelomic spread with distant contamination within the abdomen may occur. The goal of ‘wide excision' is unlikely to be achievable in most cases. Here, the objective is “planned marginal excision”, achieving appropriate margins that balance tumour control with minimising operative morbidity and retaining function. However, multi-visceral resection may be appropriate if this is necessary to permit “en bloc” resection of tumour, organs frequently sacrificed include kidney and spleen, and partial organ resection and vascular reconstructions may occasionally be required. The role of post operative radiotherapy is less well defined, and although it may be of value in individual patients, it is not considered routine as for limb sarcomas. It is often difficult to define the radiation volume and dose is limited due to the risk of small bowel and other organ toxicity. In cases where it is possible to define “high risk margins” postoperative radiotherapy to a dose of 45–50 Gy in 1.8 Gy fractions should be considered [58]. In certain situations, for example, low pelvic tumours, higher doses of radiation may be given as normal tissue tolerance is greater. Pre-operative radiotherapy may be a preferred option as the treatment volume is smaller and better defined and the tumour acts as its own “spacer” [59].

There is currently no evidence to support the use of neo-adjuvant or adjuvant chemotherapy in the management of retroperitoneal sarcomas. Palliative chemotherapy should be considered for the same indications as limb sarcomas but well-differentiated/de-differentiated liposarcoma is generally not very chemosensitive.

## 11. Borderline Tumours

### 11.1. Dermatofibrosarcoma Protuberans

DFSP is a rare neoplasm of the dermis layer of the skin. This is best considered as a borderline malignancy that rarely metastasises but is locally aggressive, may produce significant morbidity, and occasionally proves fatal. Local recurrence following surgery is common and wide excision is essential except in situations where wide excision would result in significant morbidity or functional loss. In this instance, Mohs surgery should be considered as an option if expertise in this technique is available. 

Systemic treatment is appropriate in selected cases with unresectable or metastatic disease. DFSP is driven by a t(17; 22) translocation that results in over-expression of platelet derived growth factor beta (PDGF*β*). Therefore, the PDGF*β* receptor may be inhibited by imatinib which is licensed for the treatment of unresectable DFSP.

Radiotherapy should be considered if surgery is not possible, and can result in durable remissions.

**Key Recommendations:**

- Treatment of DFSP is wide surgical excision, Mohs surgery can also be used to reduce functional loss.
- Imatinib may provide effective palliation for patients with unresectable DFSP.

### 11.2. Fibromatosis (Desmoid Tumour)

Fibromatosis is a benign, clonal tumour which, although it may be locally aggressive (even fatal on occasion), has not been reported to metastasise. Although usually sporadic it may occur in association with familial adenomatous polyposis (FAP) in which case it is termed Gardner's syndrome and is linked to germline mutations in the *APC* gene. Sporadic cases of fibromatosis are commonly liked to mutations in *CTNNB1*, the gene for beta-catenin.

The standard treatment of fibromatosis is complete resection. However the behaviour of this condition is unpredictable, in that the disease may recur locally in patients with clear surgical margins but, conversely, is not inevitable in cases where the margins were involved. Radiotherapy may be effective therapy for patients with unresectable tumours or may be given as adjuvant therapy following surgery for recurrent disease, especially if further surgery would result in significant morbidity and functional deficit. A dose of 50–54 Gy is usually employed. It should be noted that fibromatosis may undergo spontaneous stabilisation and a period of observation is appropriate for patients without significant symptoms from their disease [60].

Systemic treatment is recommended in selected cases with unresectable disease. Hormone therapies such as tamoxifen have been reported to be beneficial but, because of the unpredictable natural history of this disease, their true value remains unproven due to the lack of appropriate clinical trial data. Nonsteroidal anti-inflammatory drugs (NSAIDs) have been reported to improve the response to tamoxifen. The precise choice of NSAID is uncertain and although selective COX2 inhibitors have been used, the evidence that they are superior is lacking. NSAIDS have an impact on the beta catenin signalling pathway. Nuclear localisation of beta-catenin, the active form, is an important defining histological diagnostic criterion.

Chemotherapy is usually reserved for patients with significant symptoms who have failed to respond to more benign interventions such as the use of NSAIDs and tamoxifen. Weekly administration of methotrexate and vinblastine has reasonable activity and is generally well tolerated. More recently pegylated liposomal doxorubicin (Caelyx) has been reported to have significant activity with acceptable toxicity, and currently is considered treatment of choice by many investigators [61]. Targeted therapies such as imatinib have also been investigated and both objective remissions and disease stabilisation have been reported [62].

## Figures and Tables

**Table 1:** FNCLCC histological grading criteria [19].

Tumour differentiation	Necrosis	Mitotic count (*n*/10 high power fields)
1: well	0: absent	1: *n* < 10
2: moderate	1: <50%	2: 10–19
3: poor (anaplastic)	2: ≥50%	3: *n* ≥ 20

The sum of the scores of the three criteria determines the grade of malignancy. Grade 1: 2, 3; Grade 2: 4, 5; Grade 3: 6, 7, 8.

**Table 2:** AJCC TNM Classification for STS [21].

Classification	Description
Primary Tumour (T)	
TX	Primary tumour cannot be assessed
T0	No evidence of primary tumour
T1	Tumour ≤5 cm in greatest dimension
	T1a Superficial tumour
	T1b Deep tumour
T2	Tumour >5 cm in greatest dimension
	T2a Superficial tumour
	T2b Deep tumour

Regional lymph nodes (N)	
NX	Regional lymph nodes cannot be assessed
N0	No regional lymph node metastasis
N1	Regional lymph node metastasis

Distant metastasis (M)	
MX	Distant metastasis cannot be assessed
M0	No distant metastasis
M1	Distant metastasis

Histologic grade (G)*	
GX	Grade cannot be assessed
G1	Well-differentiated
G2	Moderately differentiated
G3	Poorly differentiated
G4	Poorly differentiated or undifferentiated

Physical examination, diagnostic radiology and biopsy provide the AJCC criteria input data needed to stage STS.

*The AJCC system uses 4 histologic grades whilst the recommended UK system (FNCLCC) uses 3. The matching grades are G1 = low grade, G2 = intermediate grade and G3 and G4 = high grade.

**Table 3:** Suggested managment of extremity STS - adapted from ESMO guidance.

Adultsoft tissue sarcoma	Standard	Individualized	Investigational
Extremities or superficial trunk			
Primary, low-grade, superficial	Surgery: wide excision		
Primary, low-grade, deep and ≤5 cm	Surgery: wide excision		
Primary, low-grade, deep and >5 cm	Surgery: wide excision ± adjuvant radiation therapy		
Primary, high-grade, superficial	Surgery: wide excision		
Primary, high-grade, deep, ≤5 cm	Surgery: wide excision	Surgery: wide excision + adjuvant radiation therapy *OR* compartmental resection	
Primary, high-grade, deep, >5 cm	Surgery: wide excision + adjuvant radiation therapy (pre *and/OR* post)	Surgery: wide excision + adjuvant radiation therapy (pre *and/OR* post) + Discussion of adjuvant Chemotherapy	Neoadjuvant chemotherapy ± postoperative chemotherapy *followed by* Wide surgical excision + adjuvant postoperative (or preoperative) radiation therapy
	Compartmental resection	Compartmental resection + Discussion of adjuvant Chemotherapy	
Local recurrence, low-grade	Surgery: wide excision + adjuvant radiation therapy (pre *and/OR* post)	Surgery: wide excision	Isolated limb perfusion
Local recurrence, high-grade	Surgery: wide excision + adjuvant radiation therapy	Surgery: wide excision + Adjuvant Radiation therapy + Discussion of Adjuvant Chemotherapy	Isolated limb perfusion
	Surgery: compartmental resection	Surgery: compartmental resection + Discussion of Adjuvant Chemotherapy	

**Table 4:** Soft tissue sarcomas grouped by chemosensitivity.

Relative chemosensitivity	Examples of soft tissue sarcomas
Chemotherapy integral to management	(i) Ewing's sarcoma family tumours
	(ii) Embryonal and alveolar rhabdomyosarcoma

Chemosensitive	(i) Synovial sarcoma
	(ii) Myxoid/round cell liposarcoma
	(iii) Uterine leiomyosarcoma

Moderately chemosensitive	(i) Pleomorphic liposarcoma
	(ii) Myxofibrosarcoma
	(iii) Epithelioid sarcoma
	(iv) Pleomorphic rhabdomyosarcoma
	(v) Leiomyosarcoma
	(vi) Malignant peripheral nerve sheath tumour
	(vii) Angiosarcoma
	(viii) Desmoplastic small round cell tumour
	(ix) Scalp and face angiosarcoma

Relatively chemo-insensitive	(i) Dedifferentiated liposarcoma
	(ii) Clear cell sarcoma
	(iii) Endometrial stromal sarcoma

Chemoinsensitive	(i) Alveolar soft part sarcoma
	(ii) Extraskeletal myxoid chondrosarcoma

## Appendix

### Key Recommendations

Clinical Presentation

1. Any patient with a soft tissue mass that is increasing in size, has a size >5cm or is deep to the deep fascia, whether or not it is painful, should be referred to a diagnostic centre with a suspected soft tissue sarcoma (STS).

Investigation

1. Magnetic resonance imaging (MRI) and core needle biopsy are recommended prior to definitive surgery.
2. Imaging of the thorax by CT scan for lung metastases should be done prior to radical treatment.
3. All patients with a suspected STS should be managed by a specialist Sarcoma multidisciplinary team (MDT) as specified in the NICE guidance.

Management

1. Surgery is the standard treatment for all patients with localised STS.
2. For those patients with resectable disease, a wide excision is the standard surgical procedure.
3. The definition of “wide” remains unclear but most would accept that an intact fascial layer or 1cm of normal tissue would be considered adequate.
4. Where a wide excision is not possible due to anatomical constraints, a planned marginal excision plus radiotherapy may be an appropriate means of achieving tumour control while maintaining function.
5. Occasionally amputation is the only surgical option to achieve adequate margins.
6. For patients with borderline resectable tumours, pre-operative treatment with chemotherapy or radiotherapy should be considered dependant on individual histology.
7. Isolated limb perfusion may permit limb salvage in some cases where amputation is the only other option.
8. Post-operative radiotherapy is recommended following surgical resection of the primary tumour for the majority of patients with high-grade tumours, and for selected patients with large or marginally excised, low-grade tumours.
9. The recommended dose for postoperative radiotherapy is 60–66 Gray (Gy); in 2 Gy per fraction.
10. Pre-operative radiotherapy is advantageous in terms of long-term functional outcome with equivalent rates of disease control when compared with postoperative radiotherapy. There is however an increased risk of postoperative wound complications.
11. The recommended dose for pre-operative radiotherapy is 50 Gy; in 2 Gy per fraction.
12. Adjuvant chemotherapy is not routinely recommended but could be considered in situations where it may contribute to local disease control, for example, where proximity to sensitive vital structures precludes giving an adequate dose of radiotherapy or in the case of an R1 resection and a further wide excision cannot be performed.

Prognosis & Follow Up

1. It is recommended that patients with intermediate or high grade sarcoma are followed up every 3-4 months for the first 2-3 years, then twice a year for up to 5 years, and annually thereafter.
2. Patients with low-grade sarcoma should be followed up every 4–6 months for 3–5 years, then annually.
3. Standard follow-up practice should consist of:
  - Investigation of any symptoms reported by the patient.
  - Clinical examination to focus on local recurrence, with imaging follow-up where indicated by clinical suspicion.
  - Routine chest x-ray to exclude pulmonary metastases.

Treatment of Advanced Disease

1. Systemic treatments for the majority of advanced STS are not curative; median survival time is ≤12 months from diagnosis of metastases.
2. Published chemotherapy response rates vary enormously; from 10–50% depending on the drugs used, patient selection, tumour grade and histological subtype.
3. Standard first-line treatment is single agent doxorubicin 75 mg/m^2^ every 3 weeks.
4. Ifosfamide at a dose of 9-10 g/m^2^ may be used first line if anthracyclines are contraindicated and may be an option for second line therapy, other options could be considered according to the histology.
5. Although the combination of doxorubicin and ifosfamide has not been demonstrated to improve survival in comparison to single agent doxorubicin first line, response rates are higher and it may be considered in individual patients.
6. Additional second-line agents include dacarbazine, trabectedin and the combination of gemcitabine + docetaxel. Reported response rates are in the range of 5–25%. Some appear more active in certain histological subtypes, particularly leiomyosarcomas and liposarcomas. The choice of agent depends on histology, toxicity profile and patient preference.
7. Surgical resection of local recurrence and pulmonary metastases should be considered in individual patients although there are limited data on survival benefit.

Specific Recommendations for Individual Histological Diagnosis

1. Standard treatment for all localised uterine sarcomas is total abdominal hysterectomy (TAH). Lymphadenectomy is not routinely indicated.
2. Oophorectomy is indicated for endometrial stromal sarcoma. These patients should not have post-operative hormone replacement therapy. Use of adjuvant anti-oestrogen therapy is not routinely indicated.
3. Adjuvant pelvic radiotherapy has not been shown to improve local control or survival, and is not routinely indicated in International Federation of Gynecology and Obstetrics (FIGO) stage I and II disease. However, it could be considered for selected high risk cases.
4. Advanced/metastatic leiomyosarcomas (LMS) and undifferentiated endometrial sarcomas are treated systemically with the same drugs as STS at other sites. Gemcitabine and docetaxel may be particularly active for LMS.
5. Advanced/metastatic endometrial stromal sarcomas (ESS) can be treated with anti-oestrogen therapy, with an aromatase inhibitor or progestagen.
6. Treatment of dermatofibrosarcoma protuberans (DFSP) is wide surgical excision, Mohs surgery can also be used to reduce functional loss.
7. Imatinib may provide effective palliation for patients with unresectable DFSP.

## References

[1] NCCN Clinical Practice Guidelines in Oncology Soft Tissue Sarcoma. Version 2.2008.

[2] Casali PG, Jost L, Sleijfer S (2008). Soft tissue sarcomas: ESMO clinical recommendations for diagnosis, treatment and follow-up. *Annals of Oncology*.

[3] NICE Guidance on Cancer Studies Improving Outcomes for People with Sarcoma.

[4] National Institute for Health and Clinical Excellence (NICE) (2005). Improving outcomes with children and young people with cancer.

[5] (2004). Guidelines for the management of gastrointestinal stromal tumours (GISTs).

[6] Jemal A, Siegel R, Ward E (2007). Cancer statistics, 2007. *CA: A Cancer Journal for Clinicians*.

[7] Albritton K, Bleyer WA (2003). The management of cancer in the older adolescent. *European Journal of Cancer*.

[8] Birch JM, Alston RD, Quinn M (2003). Incidence of malignant disease by morphological type, in young persons aged 12–24 years in England, 1979–1997. *European Journal of Cancer*.

[9] Geraci M, Birch JM, Alston RD (2007). Cancer mortality in 13 to 29-year-olds in England and Wales, 1981–2005. *British Journal of Cancer*.

[10] Ferrari A, Bleyer A (2007). Participation of adolescents with cancer in clinical trials. *Cancer Treatment Reviews*.

[11] Coindre J-M, Terrier P, Guillou L (2001). Predictive value of grade for metastasis development in the main histologic types of adult soft tissue sarcomas: a study of 1240 patients from the French Federation of Cancer Centers sarcoma group. *Cancer*.

[12] Kotilingam D, Lev DC, Lazar AJF (2006). Staging soft tissue sarcoma: evolution and change. *CA: A Cancer Journal for Clinicians*.

[13] Rasmussen SA, Friedman JM (2000). NF1 gene and neurofibromatosis 1. *American Journal of Epidemiology*.

[14] Chen CS, Suthers G, Carroll J (2003). Sarcoma and familial retinoblastoma. *Clinical and Experimental Ophthalmology*.

[15] Bell DW, Varley JM, Szydlo TE (1999). Heterozygous germ line hCHK2 mutations in Li-Fraumeni syndrome. *Science*.

[16] Johnson CJD, Pynsent PB, Grimer RJ (2001). Clinical features of soft tissue sarcomas. *Annals of the Royal College of Surgeons of England*.

[17] Christie-Large M, James SLJ, Tiessen L (2008). Imaging strategy for detecting lung metastases at presentation in patients with soft tissue sarcomas. *European Journal of Cancer*.

[18] Fletcher CDM, Unni KK, Mertens F (2002). *Pathology and Genetics of Tumours of Soft Tissue and Bone*.

[19] Italiano A, Delva F, Brouste V (2009). Effect of adjuvant chemotherapy on survival in FNCLCC grade 3 soft tissue sarcomas: a multivariate analysis of the French Sarcoma Group database. *Journal of Clinical Oncology*.

[20] Enneking WF, Spanier SS, Goodman M (1980). A system for the surgical staging of musculoskeletal sarcoma. *Clinical Orthopaedics and Related Research*.

[21] (2002). *AJCC Cancer Staging Manual*.

[22] Gerrand CH, Wunder JS, Kandel RA (2001). Classification of positive margins after resection of soft-tissue sarcoma of the limb predicts the risk of local recurrence. *Journal of Bone and Joint Surgery. British Volume*.

[23] Alektiar KM, Velasco J, Zelefsky MJ (2000). Adjuvant radiotherapy for margin-positive high-grade soft tissue sarcoma of the extremity. *International Journal of Radiation Oncology Biology Physics*.

[24] Eggermont AMM, de Wilt JHW, ten Hagen TLM (2003). Current uses of isolated limb perfusion in the clinic and a model system for new strategies. *The Lancet Oncology*.

[25] Hayes AJ, Neuhaus SJ, Clark MA (2007). Isolated limb perfusion with melphalan and tumor necrosis factor *α* for advanced melanoma and soft-tissue sarcoma. *Annals of Surgical Oncology*.

[26] Yang JC, Chang AE, Baker AR (1998). Randomized prospective study of the benefit of adjuvant radiation therapy in the treatment of soft tissue sarcomas of the extremity. *Journal of Clinical Oncology*.

[27] http://www.vortex.bham.ac.uk/VORTEX_Protocol_version_5.1__18.05.2009.pdf.

[28] Fein DA, Lee WR, Lanciano RM (1995). Management of extremity soft tissue sarcomas with limb-sparing surgery and postoperative irradiation: do total dose, overall treatment time, and the surgery-radiotherapy interval impact on local control?. *International Journal of Radiation Oncology Biology Physics*.

[29] Mundt AJ, Awan A, Sibley GS (1995). Conservative surgery and adjuvant radiation therapy in the management of adult soft tissue sarcoma of the extremities: clinical and radiobiological results. *International Journal of Radiation Oncology Biology Physics*.

[30] O’Sullivan B, Davis AM, Turcotte R (2002). Preoperative versus postoperative radiotherapy in soft-tissue sarcoma of the limbs: a randomised trial. *The Lancet*.

[31] Zagars GK, Ballo MT, Pisters PWT (2003). Preoperative vs. postoperative radiation therapy for soft tissue sarcoma: a retrospective comparative evaluation of disease outcome. *International Journal of Radiation Oncology Biology Physics*.

[32] Davis AM, O’Sullivan B, Bell RS (2002). Function and health status outcomes in a randomized trial comparing preoperative and postoperative radiotherapy in extremity soft tissue sarcoma. *Journal of Clinical Oncology*.

[33] Davis AM, O’Sullivan B, Turcotte R (2005). Late radiation morbidity following randomization to preoperative versus postoperative radiotherapy in extremity soft tissue sarcoma. *Radiotherapy and Oncology*.

[34] Tierney JF (1997). Adjuvant chemotherapy for localised resectable soft-tissue sarcoma of adults: Meta-analysis of individual data. *Lancet*.

[35] (2000). Adjuvant chemotherapy for localised respectable soft tissue sarcoma in adults: sarcoma meta-analysis collaboration (SMAC). *Cochrane Database of Systematic Reviews*.

[36] Pervaiz N, Colterjohn N, Farrokhyar F (2008). A systematic meta-analysis of randomized controlled trials of adjuvant chemotherapy for localized resectable soft-tissue sarcoma. *Cancer*.

[37] Frustaci S, Gherlinzoni F, De Paoli A (2001). Adjuvant chemotherapy for adult soft tissue sarcomas of the extremities and girdles: results of the italian randomized cooperative trial. *Journal of Clinical Oncology*.

[38] Woll PJ, van Glabbeke M, Hohenberger P (2007). Adjuvant chemotherapy with doxorubicin and ifosfamide in resected soft tissue sarcoma: interim analysis of a randomised phase III trial. *Journal of Clinical Oncology*.

[39] Salgado R, van Marck E, De Schepper AM, Vanhoemacker F, Gielen J (2006). Soft tissue tumours: the surgical pathologist’s perspective. *Imaging of Soft Tissue Tumors*.

[40] Spurrell EL, Fisher C, Thomas JM (2005). Prognostic factors in advanced synovial sarcoma: an analysis of 104 patients treated at the Royal Marsden Hospital. *Annals of Oncology*.

[41] Karavasilis V, Seddon B, Ashley S (2006). Significant clinical benefit of first-line palliative chemotherapy in advanced soft-tissue sarcoma. *Journal of Clinical Oncology*.

[42] Jones RL, Fisher C, Al-Muderis O (2005). Differential sensitivity of liposarcoma subtypes to chemotherapy. *European Journal of Cancer*.

[43] Grobmyer SR, Brennan MF (2003). Predictive variables detailing the recurrence rate of soft tissue sarcomas. *Current Opinion in Oncology*.

[44] Gerrand CH, Billingham LJ, Woll PJ (2007). Follow up after primary treatment of soft tissue sarcoma: a survey of current practice in the United Kingdom. *Sarcoma*.

[45] Blay J-Y, van Glabbeke M, Verweij J (2003). Advanced soft-tissue sarcoma: a disease that is potentially curable for a subset of patients treated with chemotherapy. *European Journal of Cancer*.

[46] van Glabbeke M, van Oosterom AT, Oosterhuis JW (1999). Prognostic factors for the outcome of chemotherapy in advanced soft tissue sarcoma: an analysis of 2,185 patients treated with anthracycline- containing first-line regimens—an European organization for research and treatment of cancer soft tissue and bone sarcoma group study. *Journal of Clinical Oncology*.

[47] Bramwell VH, Anderson D, Charette ML (2003). Doxorubicin-based chemotherapy for the palliative treatment of adult patients with locally advanced or metastatic soft tissue sarcoma. *Cochrane Database of Systematic Reviews*.

[48] van Oosterom AT, Mouridsen HT, Nielsen OS (2002). Results of randomised studies of the EORTC Soft Tissue and Bone Sarcoma Group (STBSG) with two different ifosfamide regimens in first- and second-line chemotherapy in advanced soft tissue sarcoma patients. *European Journal of Cancer*.

[49] Palumbo R, Palmeri S, Antimi M (1997). Phase II study of continuous-infusion high-dose ifosfamide in advanced and/or metastatic pretreated soft tissue sarcomas. *Annals of Oncology*.

[50] Buesa JM, López-Pousa A, Martín J (1998). Phase II trial of first-line high-dose ifosfamide in advanced soft tissue sarcomas of the adult: a study of the Spanish Group for Research on Sarcomas (GEIS). *Annals of Oncology*.

[51] http://www.cancer.gov/clinicaltrials/EORTC-62012.

[52] Leu KM, Ostruszka LJ, Shewach D (2004). Laboratory and clinical evidence of synergistic cytotoxicity of sequential treament with gemcitabine followed by docetaxel in the treatment of sarcoma. *Journal of Clinical Oncology*.

[53] Maki RG, Wathen JK, Patel SR (2007). Randomized phase II study of gemcitabine and docetaxel compared with gemcitabine alone in patients with metastatic soft tissue sarcomas: results of sarcoma alliance for research through collaboration study. *Journal of Clinical Oncology*.

[54] Demetri GD, Chawla SP, von Mehren M (2009). Efficacy and safety of trabectedin in patients with advanced or metastatic liposarcoma or leiomyosarcoma after failure of prior anthracyclines and ifosfamide: results of a randomized phase II study of two different schedules. *Journal of Clinical Oncology*.

[55] Skubitz KM, Haddad PA (2005). Paclitaxel and pegylated-liposomal doxorubicin are both active in angiosarcoma. *Cancer*.

[56] Schlemmer M, Reichardt P, Verweij J (2008). Paclitaxel in patients with advanced angiosarcomas of soft tissue: a retrospective study of the EORTC soft tissue and bone sarcoma group. *European Journal of Cancer*.

[57] Chu MC, Mor G, Lim C (2003). Low-grade endometrial stromal sarcoma: hormonal aspects. *Gynecologic Oncology*.

[58] Feng M, Murphy J, Griffith KA (2007). Long term outcomes after radiotherapy for retroperitoneal and deep truncal sarcoma. *International Journal of Radiation Oncology Biology Physics*.

[59] Tzeng C-WD, Fiveash JB, Popple RA (2006). Preoperative radiation therapy with selective dose escalation to the margin at risk for retroperitoneal sarcoma. *Cancer*.

[60] Phillips SR, A’Hern R, Thomas JM (2004). Aggressive fibromatosis of the abdominal wall, limbs and limb girdles. *British Journal of Surgery*.

[61] Constantinidou A, Scurr M, Jones R (2009). Treatment of aggressive fibromatosis with pegylated liposomal doxorubicin; the Royal Marsden Hospital experience. *Journal of Clinical Oncology*.

[62] Dufresne A, Penel S, Salas S (2009). Updated outcome with long-term follow-up of imatinib for the treatment of progressive or recurrent aggressive fibromatosis (desmoids tumor): a FNCLCC/French Sarcoma Group phase II trial. *Journal of Clinical Oncology*.

